# *Bid*-deficient fish delay grass carp reovirus (GCRV) replication and attenuate GCRV-triggered apoptosis

**DOI:** 10.18632/oncotarget.19460

**Published:** 2017-07-22

**Authors:** Libo He, Hao Wang, Lifei Luo, Yongming Li, Rong Huang, Lanjie Liao, Zuoyan Zhu, Yaping Wang

**Affiliations:** ^1^ State Key Laboratory of Freshwater Ecology and Biotechnology, Institute of Hydrobiology, Chinese Academy of Sciences, Wuhan 430072, China; ^2^ University of Chinese Academy of Sciences, Beijing 100049, China; ^3^ Department of Microbiology and Immunology, Albert Einstein College of Medicine, Bronx, NY 10461, USA

**Keywords:** Bid, grass carp reovirus, grass carp, rare minnow, apoptosis

## Abstract

Bid, BH3-interacting domain death agonist, is a pro-apoptotic BH3-only member of Bcl-2 family, playing an important role in apoptosis. In the study, *Bid* genes from grass carp (*Ctenopharyngodon idellus*) and rare minnow (*Gobiocypris rarus*), named *CiBid* and *GrBid*, were cloned and analyzed. *Bid* was constitutively expressed in all examined tissues of grass carp, but the expression level varied in different tissues. Following grass carp reovirus (GCRV) stimulation *in vivo*, *Bid* and apoptosis related genes *Caspase-9* and *Caspase-3* was up-regulated significantly at the late stage of infection. Moreover, we generated a *Bid*-deficient rare minnow (*Bid*^-/-^) to investigate the possible role of *Bid* in GCRV-triggered apoptosis. We found that the survival time of *Bid*^-/-^ rare minnow after GCRV infection was extended when compared with wild-type fish, the relative copy number of GCRV in *Bid*^-/-^ rare minnow was lower than that in wild-type fish, and the expression level of *Caspase-9* and *Caspase-3* in *Bid*^-/-^ rare minnow were significantly lower than that in the wild-type fish. Collectively, the current data revealed the important role of *Bid* during virus-induced apoptosis in teleost fish. Our study would provide new insight into understanding the GCRV induced apoptosis and may provide a target gene for virus-resistant breeding in grass carp.

## INTRODUCTION

The grass carp (*Ctenopharyngodon idellus*) is one of the most famous aquaculture species worldwide, accounting for 13% of global freshwater aquaculture production in 2014 [1, 2]. However, frequent diseases always result in huge economic loss to the grass carp cultivation industry. Of these diseases, grass carp hemorrhage disease caused by grass carp reovirus (GCRV) has been being noticed with special concern by fish breeding scientists with the hope of disease-resistant breeding. GCRV, belonging to the family *Reoviridae*, genus *Aquareovirus* [3], is a double-stranded RNA (dsRNA) virus reported mainly in China and could trigger apoptosis in *C. idellus* kidney (CIK) cells [4]. However, the mechanism of apoptosis induced by GCRV, which is critical for the defense of GCRV and virus-resistant breeding, remains unknown. The rare minnow (*Gobiocypris rarus*), a Chinese native species belonging to the family *Cyprinidae*, could be infected by GCRV, resulting in an up to 100% mortality rate [5]. Moreover, due to its propagational biological characteristics, rare minnow has the potential to be used as a model fish in aquatic toxicity testing, chemical safety assessment, and virus-resistant breeding [6].

In CIK cells, both death receptor pathway and mitochondrial pathway were involved in GCRV-induced apoptosis [4]. Bid, BH3-interacting domain death agonist, is a pro-apoptotic BH3-only member of Bcl-2 family, serving as a key link in the amplification of various apoptosis signals other than the death receptor pathway [7, 8]. In the mitochondrial pathway, caspase-8 activated by death receptors pathway cleaves Bid and the truncated Bid (tBid) then translocates to the mitochondria. The BH3 domain of tBid can interact with pro-apoptotic Bcl-2 proteins, including Bax and Bak, to mediate the mitochondrial outer membrane permeabilization (MOMP), leading the release of cytochrome c (cyt c) into cytosol with subsequent activation of apoptosis [7–13].

Interestingly, numerous studies in animal models demonstrated that Bid could be viewed as potential therapeutic target for some certain diseases. Suppression of hepatocyte *Bid* using an antisense approach could not only effectively attenuate the hepatocytes apoptosis induced by Glychochenodeoxycholate (GCDC), but also ameliorate liver injury in a mice model of extrahepatic cholestasis [14]. In hepatocellular-specific *Bid* deficient mice, their liver showed strong resistance to hepatocellular apoptosis and hepatotoxicity in comparison with controls, suggesting inhibition of *Bid* was critical for the resistance to the lethal effects of Fas activation *in vivo* [15]. In contrast to controls, an upregulation of *Bid* could be detected in septic shock patients, indicating pro-apoptotic gene *Bid* has great potential as a biomarker to monitor sepsis [16]. *Bid*^-/-^ mice showed no lung injury after Lipopolysaccharides (LPS) stimulation [17]. Survival of *Bid*-deficient mice was significantly increased when compared with wild type mice after reovirus infection [7, 18]. However, there is limited knowledge of *Bid* in teleost fish, and the specific role of *Bid* during the virus-induced apoptosis in teleost fish is still unclear.

This study examined the mechanisms of GCRV-induced apoptosis and investigated the possibility that knocking out of *Bid* may provide an innovative strategy for enhancing survival of rare minnow following virus infection. In the present study, *Bid* genes from grass carp (*CiBid*) and rare minnow (*GrBid*) were cloned and analyzed. The expression pattern of *CiBid* among different tissues and the response to GCRV were studied *in vivo*. Furthermore, we examined the function of *Bid* using genetically deficient (*Bid*^-/-^) rare minnow. Our results suggested that the replication of GCRV and virus-triggered apoptosis were both suppressed in *Bid*^-/-^ rare minnow in comparison with wild-type rare minnow. Our study provides new insight into understanding the GCRV induced apoptosis and suggests that *Bid* may be a target gene for virus-resistant breeding in grass carp.

## RESULTS

### GCRV induced apoptosis in infected fish

**Figure 1:**
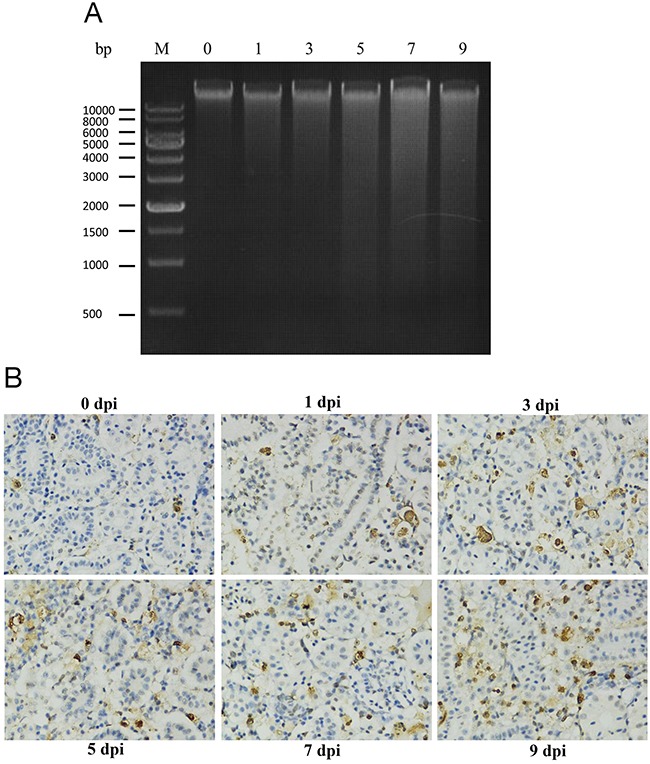
GCRV induced apoptosis in grass carp **(A)** DNA ladder assay confirmed the apoptosis in grass carp induced by GCRV. Spleen samples were collected from grass carp before (0 day) and after (1, 3, 5, 7, and 9 days) GCRV infection. DNA was extracted and subjected to electrophoresis in 1% agarose gel. M: DNA molecular weight marker. **(B)** TUNEL assay confirmed the GCRV induced apoptosis in grass carp. Kidney samples were collected from grass carp before (0 day) and after (1, 3, 5, 7, and 9 days) GCRV infection and subjected to TUNEL assay. The blue stains indicate the normal cells while the brown stains represent the apoptotic cells. The figure showed the apoptosis rate was increased after GCRV infection. Magnification: 400×.

Spleen tissues were randomly collected at 0 day before GCRV infection or at 1, 3, 5, 7, and 9 days post infection (dpi). DNA isolated from the spleens were then subjected to agarose gel electrophoresis. Apoptosis is characterized by the activation of endogenous endonuclease, resulting in the digestion of chromatin and the formation of a ladder of fragments that were multiples of approximately 200 base pairs in length. As shown in Figure 1A, no or little detectable apoptotic DNA fragmentation was visualized by the DNA laddering assay in the mock-infected sample or early stage (1 and 3 dpi) of infected samples. However, obvious apoptotic DNA fragmentation was observed at the late stage of GCRV infection (5, 7, and 9 dpi). Moreover, terminal deoxynucleotidyl transferase dUTP nick end labeling (TUNEL) assay was also performed for kidney samples that collected at the same time points as above. In Figure 1B, fish kidney showed more apoptotic cells stained with by TUNEL asssy at 5, 7, and 9 dpi, identifying severe apoptosis happened at the late time points. Overall, these results strongly suggested that GCRV induced apoptosis in infected fish

### Characteristics and phylogenetic analysis of *CiBid* and *GrBid* genes

The full length cDNA of *Bid* genes from grass carp and rare minnow, named *CiBid* (Genbank accession number: KX532178) and *GrBid* (Genbank accession number: KX532179), were obtained by PCR and rapid-amplification of cDNA ends (RACE). The full length of the *CiBid* was 986bp including 555bp open reading frame (ORF) that encoded a 184 amino acid protein (Supplementary Figure 1). *GrBid* cDNA was 923 bp long and included a 573 bp ORF encoding a predicted polypeptide of 190 amino acids (Supplementary Figure 2). Genomic DNA of both *CiBid* and *GrBid* genes contained four exons and three introns following the splicing consensus rule of GT/AG.

**Figure 2:**
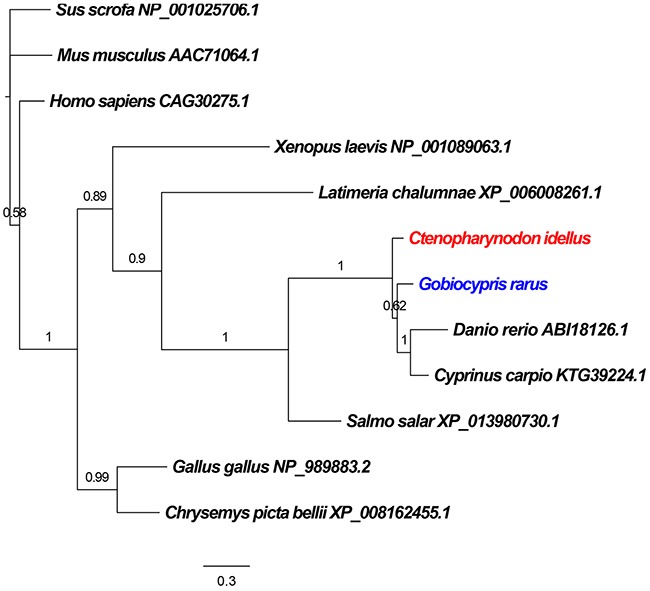
Phylogenetic relationship of the Bid proteins in different species Phylogenetic tree was constructed using MrBayes software. *Sus scrofa*, *Homo sapiens* and *Mus musculus* were introduced as outgroups. The GenBank accession numbers of Bid proteins are given after the species names in the tree.

To determine the evolutionary status of CiBid and GrBid, a phylogenetic tree was constructed based on the corresponding amino acid sequences from teleost fish (including *Latimeria chalumnae*, *Ctenopharynodon idellus*, *Gobiocypris rarus*, *Danio rerio*, *Cyprinus carpio*, and *Salmo salar*), aves (such as *Gallus gallus*), amphibia (including *Xenopus laevis* and *Chrysemys picta bellii*), and mammalia (including *Homo sapiens*, *Mus musculus*, and *Sus scrofa*). As shown in Figure 2, the result showed that Bid from the teleost fish and *X. laevis* fell into one branch; Bid from *C. picta bellii and G. gallus* fell into another branch. Bid from the mammalia were considered as an outgroup of the tree. In the tree, CiBid and GrBid were closely related to proteins from *D. rerio* and *C. carpio*.

### Expression of *CiBid* in different tissues

**Figure 3:**
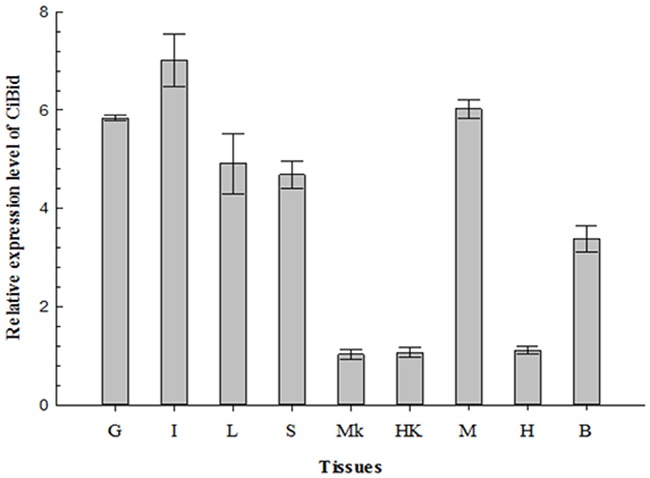
qRT-PCR analysis of the expression level of *CiBid* in different tissues of grass carp *β-actin* was used as an internal control for real-time PCR. The relative expression level was the ratio of gene expression level in different tissues relative to that in the middle kidney. G, Gill; I, Intestine; L, Liver; S, Spleen; MK, Middle Kidney; HK, Head Kidney; M, Muscle; H, Heart; B, Brain.

Real-time quantitative PCR (RT-qPCR) was performed on nine tissues from healthy grass carp (gill, intestine, liver, spleen, middle kidney, head kidney, muscle, heart, and brain) to study the expression profile of *CiBid*. As shown in Figure 3, *CiBid* was constitutively expressed in all examined tissues of grass carp, but the relative expression level differed. *CiBid* was strongly expressed in intestine, gill and muscle, comparatively highly in liver, spleen and brain, while at low levels in heart, middle kidney and head kidney (Figure 3).

### Expression analysis of *CiBid* and apoptosis-related genes after GCRV stimulation

**Figure 4:**
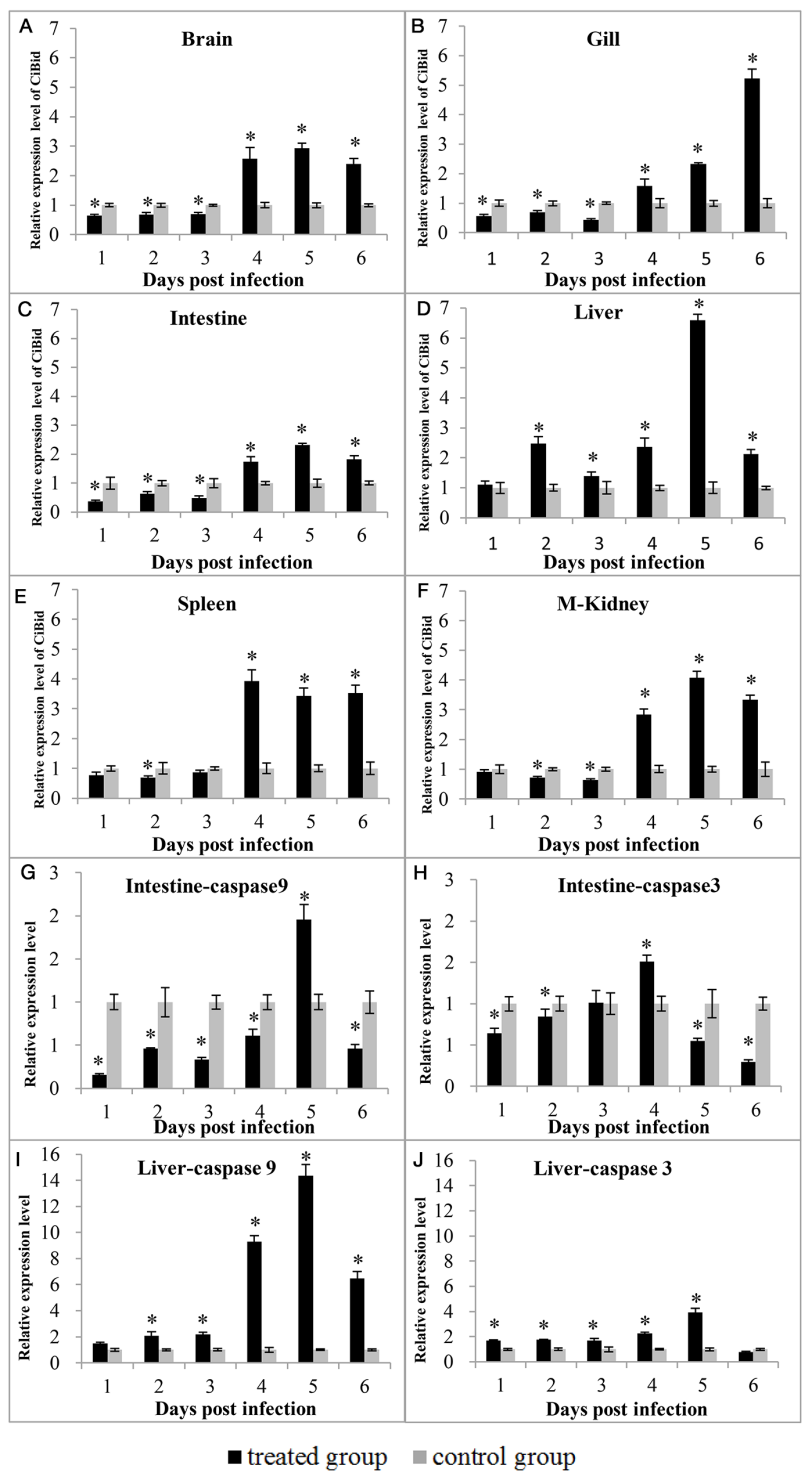
Expression patterns of *CiBid* **(A, B, C, D, E**, and **F)**, *CiCaspase-9* **(G** and **I)** *and CiCaspase-3* **(H** and **J)** in different tissues after GCRV infection. The relative expression level was the ratio of gene expression level in treated group relative to that in the negative control group in the same tissue normalized to the *β-actin* gene. Significant difference (*p*<0.05) between the control and treated group is indicated by asterisks (*).

To investigate the effect of GCRV stimulation on expression of *CiBid* and apoptosis-related genes, RT-qPCR was carried out in gill, intestine, liver, spleen, kidney, and brain of grass carp that were collected from the treated group and negative control group at different days (1, 2, 3, 4, 5, and 6) after GCRV infection. As shown in Figure 4, the expression level of *CiBid* altered significantly in all examined tissues upon GCRV stimulation (*p*<0.05). mRNA expression level of *CiBid* showed low level at the early phase (1, 2, and 3 days post infection); however, it was increased at the late phase of infection (4, 5, and 6 days post infection). The highest expression level of *CiBid* in brain, intestine, liver, and kidney was observed at the fifth day (Figure 4A, 4C, 4D, and 4F), while the expression level of *CiBid* in gill and spleen peaked at the sixth and fourth day (Figure 4B and 4E).

In addition, the expression profiles of *CiCaspase-9* and *CiCaspase-3* in liver and intestine after GCRV infection were also examined. As shown in Figure 4G, 4H, 4I, and 4J, the expression profiles of *CiCaspase-9* and *CiCaspase-3* were the same as we described previously [19]. To be specific, expression level of *CiCaspase-9* and *CiCaspase-3* in intestine was decreased at first upon GCRV stimulation and then began to up-regulate significantly around 4 or 5 days post stimulation (Figure 4G and 4H). Nevertheless, *CiCaspase-9* and *CiCaspase-3* showed a different pattern in liver. Expression level of *CiCaspase-9* and *CiCaspase-3* almost up-regulated continuously in liver and reached the peak at 4 or 5 day post infection (Figure 4I and 4J). Anyway, the up-regulation *CiCaspase-9* and *CiCaspase-3* further suggested that GCRV-induced apoptosis occurred.

### Generate *Bid*-deficient rare minnow using CRISPR/Cas9 system

**Figure 5:**
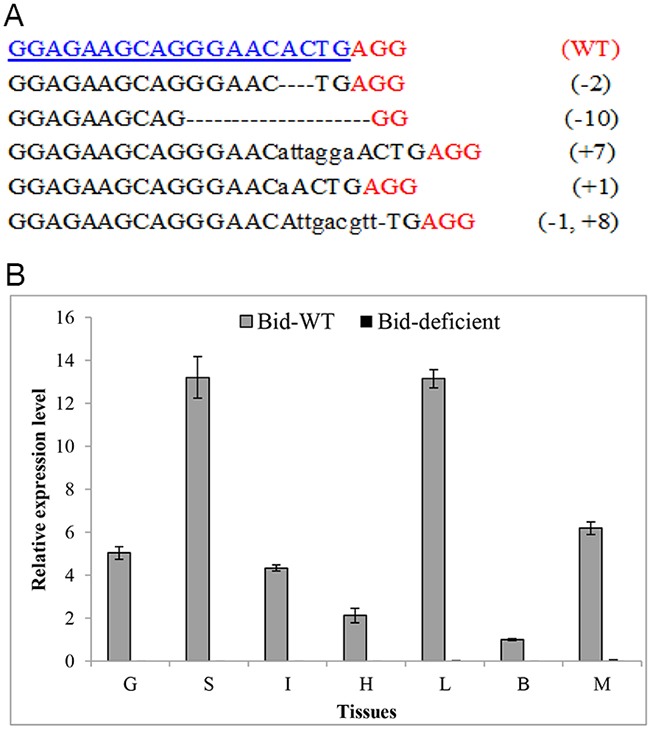
Efficient disruption of rare minnow *Bid* gene by CRISPR/Cas9 **(A)** Multiple mutations induced by CRISPR/Cas9 system in the target sequence. Numbers to the right of the targeting sequences indicated the loss or insert of bases for each allele, with the number of bases inserted (+) and deleted (-) indicated in parentheses. Deletions were shown as black dashes and insertions as lower case letters. The wild-type (WT) sequence was shown at the top with the target site highlighted in blue and the PAM sequence highlighted as red underlined text. **(B)** Confirm the loss of *Bid* expression level by RT-qPCR. *β-actin* was used as an internal control for RT-qPCR. The relative expression level was the ratio of gene expression level in different tissues relative to that in the brain tissue from the wild-type fish. G, Gill; S, Spleen; I, Intestine; H, Heart; L, Liver; B, Brain; M, Muscle.

To further investigate the possible role of *Bid* gene in fish, we chose rare minnow as a model fish to generate *Bid*-deficient line (*Bid*^-/-^) by CRISPR/Cas9 system. As previously reported, mutations caused by Cas9 were largely predictable as the induced mutant loci were produced at a fixed target sequence [20]. The target sequence of *GrBid* (5’-GGAGAAGCAGGGAACACTGA GG-3’) was located at 27bp downstream of the translation start site (ATG) of *Bid* gene in rare minnow. Thus, mutation in the target sequence would result in the loss of function. The one-cell stage rare minnow embryos were injected with Cas9 mRNA and sgRNA. PCR and sequencing results of the injected embryos showed that mutations were yielded in the *GrBid* target site (Figure 5A). The -10bp mutation was chosen for further study and the homozygous *Bid*-deficient rare minnow was obtained in finally. Moreover, RT-qPCR was carried out to confirm that CRISPR/Cas9 could induce loss-of-function mutation for Bid in rare minnow. Seven tissues (gill, spleen, intestine, heart, liver, brain, and muscle) were collected from *Bid*-deficient and wild-type fish. RNA was extracted and then reverse-transcribed to obtain cDNA for RT-qPCR. As shown in Figure 5B, no or very little expression level of *Bid* gene (CT value ≥ 33) was detected in *Bid*-deficient fish whereas expression level of *Bid* in wild-type fish was normal (CT value range from 23~26). These results further confirmed the generation of *Bid*-deficient rare minnow.

### *Bid*-deficient rare minnow delayed the death after GCRV infection

**Figure 6:**
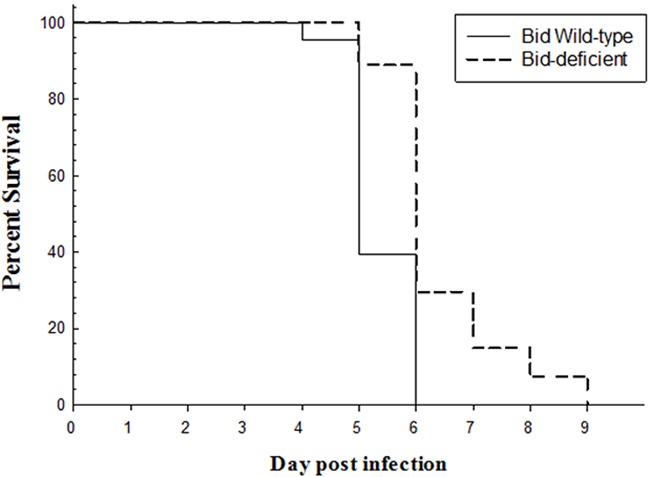
Survival curve of *Bid*-deficient and wild-type rare minnow after GCRV infection *Bid*-deficient wild-type rare minnow were immersed into GCRV solution for half an hour and transferred to aerated freshwater and cultured at 28 °C. The dead individuals in both groups were recorded every day.

Since *Bid* plays important role in virus-induced apoptosis, we tested whether *Bid*-deficient rare minnow could modulate the GCRV-induced death compared with the wild type rare minnow. As shown in Figure 6, following infection with GCRV, the wild-type rare minnow started to die at as early as the fourth day, and all of them died in 6 dpi with a median survival time of 5 days. In contrast, *Bid*-deficient rare minnow started to die at the fifth day, which continued to the ninth day post GCRV stimulation (Figure 6). Moreover, the median survival time of *Bid*-deficient rare minnow after infected with GCRV (6 days) was more than that of wild-type rare minnow (5 days). Obviously, the results revealed that *Bid*-deficient rare minnow delayed the death induced by GCRV infection.

### *Bid*-deficient rare minnow reduced the efficiency of GCRV replication

**Figure 7:**
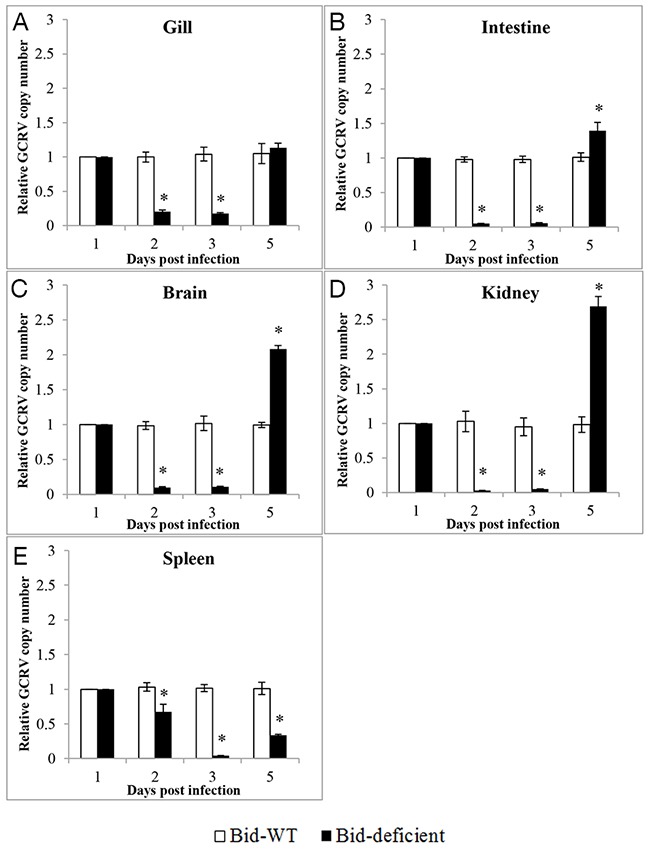
Relative copy numbers of GCRV *in vivo* After infection with GCRV for 1, 2, 3 and 5 day, total RNA was isolated to analyze the relative copy number of GCRV in gill **(A)**, intestine **(B)**, brain **(C)**, kidney **(D)** and spleen **(E)**. The relative copy number of GCRV was the ratio of GCRV S6 segment expression level in *Bid*-deficient fish relative to that in wild-type fish. Significant difference (*p*<0.05) in viral copy numbers between the samples from wild-type and *Bid*-deficient are indicated with an asterisk (*).

To determine whether the delay of death in *Bid*-deficient rare minnow group was correlated with the efficiency of GCRV replication, relative copy number of GCRV in gill, intestine, brain, kidney and spleen from GCRV-infected *Bid*-deficient rare minnow was detected and compared with that of wild-type rare minnow. As shown in Figure 7, at 2 and 3 dpi, significantly lower virus copy numbers were observed in all examined tissues from *Bid*-deficient rare minnow, compared with those in the wild-type rare minnow (*p*<0.05; Figure 7). In detail, at the second and third days after the GCRV challenge, the copy numbers of GCRV in gill, intestine, brain, and kidney of *Bid*-deficient rare minnow were ~5-fold, ~20-fold, ~10-fold, and ~20-fold lower than those in wild-type group (Figure 7A, 7B, 7C, and 7D). Virus copy numbers in spleen of wild-type rare minnow were ~2-fold at the day 2, and ~20-fold at the day 3 post-stimulation, in comparison with that in *Bid*-deficient fish (Figure 7E). However, the copy numbers of GCRV in gill, intestine, brain and kidney of *Bid*-deficient rare minnow were equivalent, or even higher than those in wild-type group at five days post infection (Figure 7). Collectively, these results indicated that the replication efficiency of GCRV decreased in the absence of *Bid* in rare minnow.

### *Bid*-deficient rare minnow attenuated GCRV-induced apoptosis

**Figure 8:**
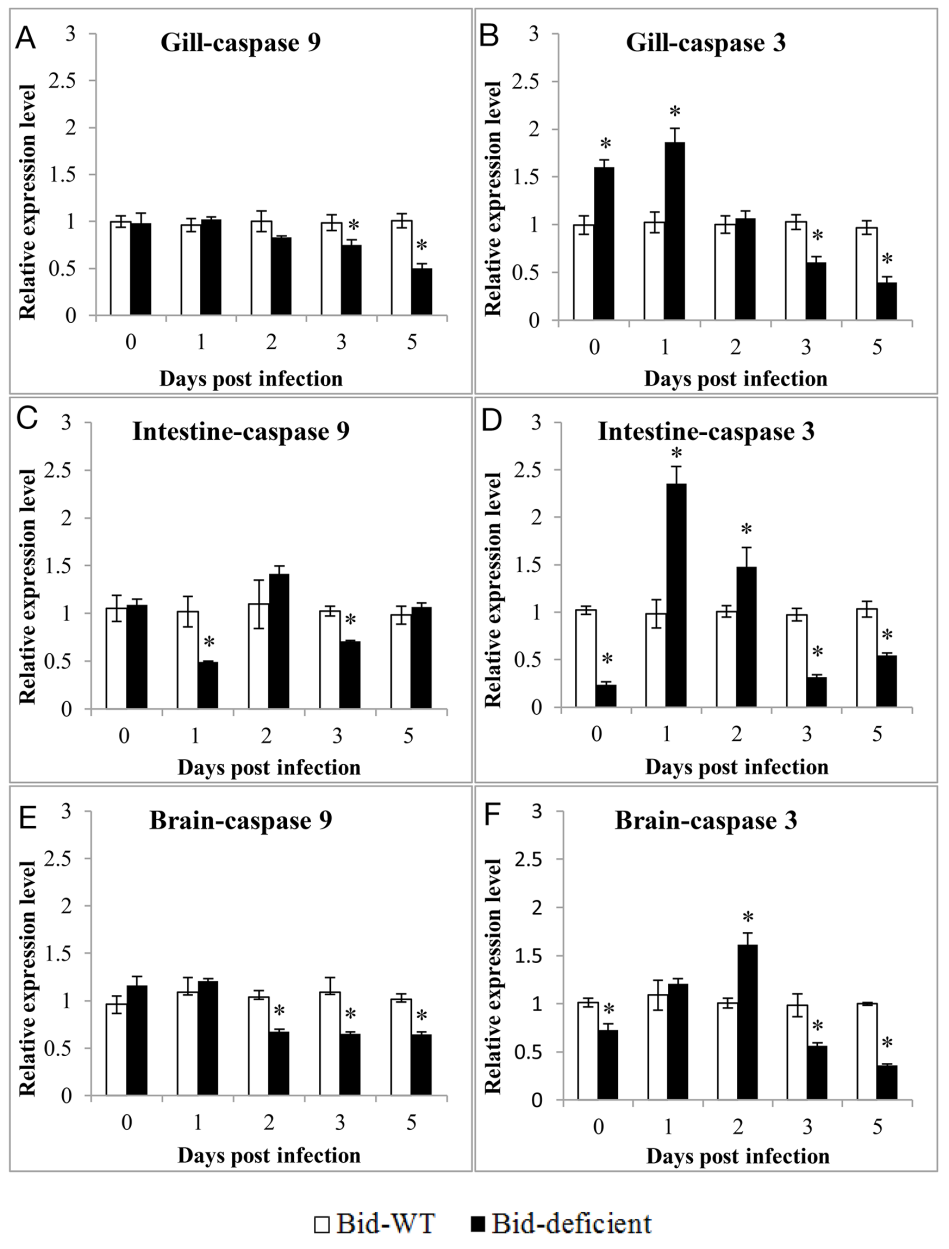
Expression profiles of *GrCaspase-9* and *GrCaspase-3* in Gill **(A & B)**, Intestine **(C & D)** and Brain **(E & F)** of *Bid*-deficient and wild-type rare minnow post GCRV infection. Transcriptional levels of *GrCaspase-9* and *-3* were detected by qRT-PCR and normalized to *Grβ-actin*. The relative expression level was the ratio of gene expression level in *Bid*-deficient fish relative to that in wild-type fish.

In order to investigate whether the delay of death in *Bid*-deficient rare minnow was attributed to the degree of virus-triggered apoptosis, the differences in expression level of apoptosis related genes between *Bid*-deficient and wild-type rare minnow were compared. As shown in Figure 8, overall, at the 3 and 5 dpi, the expression level of *GrCaspase-9* and *GrCaspase-3* in gill, intestine and brain of *Bid*-deficient rare minnow were significantly lower than those in the wild-type fish (Figure 8). Specifically, as for *GrCaspase-3*, the relative expression level in gill, intestine and brain of *Bid*-deficient rare minnow were even notably higher than these in wild-type group at 0, 1, or 2 dpi; however, the expression level were significantly lower in *Bid*-deficient rare minnow at 3 and 5 dpi (*p*<0.05; Figure 8B, 8D, and 8F). In comparison with wild-type rare minnow, the expression of *GrCaspase-9* in gill, intestine and brain of *Bid*-deficient rare minnow displayed significantly lower level at 2, 3 or 5 dpi (*p*<0.05; Figure 8A, 8C, and 8E). These data suggested that GCRV-induced apoptosis in rare minnow was diminished in the absence of *Bid*.

## DISCUSSION

Bid, a BH-3-only multi-functional molecule, plays a vital role in virus-triggered diseases. Caspase-8 activation and Bid cleavage are critical events for virus-induced apoptosis through a mitochondria-mediated pathway [21–29]. It has been demonstrated that Hepatitis C virus (HCV) core protein could mediate TRAIL-induced apoptosis by enhancing Bid cleavage and activation of mitochondria apoptosis signaling pathway [23]. In comparison to wild-type mice, *Bid*-deficient mice showed enhanced resistance to reovirus [7]. However, in teleost fish, the mechanism of Bid regulating virus-induced apoptosis signal transduction is still not elucidated. We cloned *Bid* genes from grass carp and rare minnow, examined the expression pattern before and after virus infection, and investigated the possible role using genetically deficient rare minnow.

Interestingly, the overall expression level of *Bid* was down-regulated at the early phase of infection (1, 2, and 3 dpi) whereas up-regulated at the late phase (4, 5, and 6 dpi). At the early stage of virus infection, host cell apoptosis was inhibited by virus in order to facilitate the replication and spread of virus [30]. Thus, as a pro-apoptotic protein, mRNA expression level of *Bid* was down-regulated. However, the host could respond to virus infection and cell apoptosis occurred at the late stage as a self-protection mechanism to limit the spread of progeny virus [31]. Therefore, it is not surprising that the mRNA expression level of *Bid* was up-regulated at the late stage of infection. Similar expression pattern was also observed in other pro-apoptotic genes in grass carp [19, 32]. Moreover, the dynamic changes of GCRV copy number in infected grass carp was detected in our previously study [33]. Results showed that the relative copy number of GCRV (type II GCRV) was extremely low in 1 and 3 days post-infection, followed by a dramatic increase in 5 days post stimulation and a slight reduction in 7 days post infection. Obviously, the copy number of GCRV was consistent with the expression level of *CiBid* in some degree. Overall, the dynamics of GCRV copy number was associated with the expression level of *CiBid*. After GCRV infection, the expression of *CiBid* altered significantly in all examined tissues but the response was different among these tissues. The strong response was observed in liver, spleen, and kidney tissue. Previous study suggested that tissue tropism of GCRV was existed. The liver, spleen, kidney, intestine, and muscle of fish were more susceptible to GCRV and had a higher number of viral RNA copies [34]. Thus, the strong response in liver, spleen, and kidney tissue may be caused by higher virus titers in these tissues. Not only *Bid*, but also *CiCaspase-9* and *CiCaspase-3*, were up-regulated upon GCRV infection. Previous studies have shown that both HIV-1 protease and HCV could activate procaspase-8, which was associated with subsequent events including cleavage of Bid, mitochondrial release of cytochrome c, and activation of the downstream effectors caspase-9 and caspase-3 [23, 35–37]. Hence, the up-regulation of *CiBid*, *CiCaspase-9*, and *CiCaspase-3* implied that *CiBid* contributed to GCRV-induced apoptosis.

Since rare minnow is small in size, easy to culture, adaptable to a wide temperature range, and has a relatively short life cycle, it was considered as an emerging model in fish biology [6]. Moreover, similar with grass carp, rare minnow is much more vulnerable to GCRV. Therefore, we selected rare minnow as a fish model for virus-resistant breeding program of grass carp. We successfully obtained *Bid*^-/-^ rare minnow by use of the CRISPR/Cas9 system, indicating this technique is feasible and valid in editing genes of rare minnow. In our study, following infection with GCRV, the median survival time of *Bid*^-/-^ rare minnow was significantly delayed and the duration of death was extended. *Bid*^-/-^ mice showed a certain degree of resistance to reovirus, but the resistance was in correlation with the virus dose and how it infected [7]. Therefore, we suspected that the survival rate of *Bid*-deficient rare minnow may be associated with the dose of GCRV we used. However, this hypothesis needs further confirmation. RT-qPCR showed that the copy number of virus in all examined tissues (gill, intestine, spleen, kidney, spleen, and brain) of *Bid*^-/-^ rare minnow was lower at the early stage post infection (2 and 3 dpi) compared with the wild-type fish, but equivalent or even higher at the late phase of infection (5 dpi). In *Bid*^-/-^ mouse model, the reovirus titers in intestine, liver, and heart were also lower than those in wild-type mice at the early stage of peroral inoculation, but equivalent at the late phase after infection [7]. When the mouse was infected via intracranial inoculation, there were no obvious differences in virus titers between wild-type and *Bid*^-/-^ mouse [7]. Therefore, similar to mouse, *Bid*^-/-^ rare minnow could reduce the efficiency of GCRV replication, but the reduction could be affected by virus dose.

In our study, the overall expression level of *GrCaspase-9* and *GrCaspase-3* in *Bid*^-/-^ rare minnow were lower than those in wild-type group, suggesting knocking out of *Bid* in rare minnow could effectively attenuate the degree of GCRV-induced apoptosis. Moreover, as described before, the copy number of GCRV in *Bid*^-/-^ rare minnow was lower at the early stage post infection (2 and 3 dpi) when compared with the wild-type fish. However, it is not clear whether the decreased replication efficiency of GCRV was a cause, or a result, of the reduced apoptosis. Because some virus could use apoptosis as a mechanism of virus spread; meanwhile, other viruses’ successful replication relied on the ability of certain viral products (such as E1B-19K, IE1, and SPI-2) to block or delay apoptosis [38–40]. The relationship between the decreased replication efficiency of virus and reduced apoptosis needs further study.

In conclusion, *Bid* gene was cloned from grass carp (*CiBid*) and rare minnow (*GrBid*), respectively. *CiBid* was constitutively expressed in all examined tissues of healthy grass carp, but the expression level varied in different tissues. Following GCRV stimulation *in vivo*, *CiBid* and apoptosis related genes *Caspase-9* and *Caspase-3* were up-regulated significantly. Additionally, we use *Bid*^-/-^ rare minnow as a model to investigate the possible role of *Bid* in regulating GCRV-triggered apoptosis. We found that *Bid*-deficient rare minnow delayed the GCRV replication and attenuated GCRV-induced apoptosis. Our study would provide new insight into understanding the GCRV induced apoptosis and may provide a target gene for virus-resistant breeding in grass carp.

## MATERIALS AND METHODS

### Ethics statement

Animal welfare and experimental procedures were carried out in accordance with the Guide for the Care and Use of Laboratory Animals (Ministry of Science and Technology of China, 2006), and the protocol was approved by the committee of the Institute of Hydrobiology, Chinese Academy of Sciences (CAS). All surgery was performed under eugenol anesthesia, and all efforts were made to minimize suffering.

### Experimental animals, virus exposure, and sample collection

Grass carp, averaging 10 cm in body length, were acclimatized in aerated freshwater at 28 °C and were fed twice daily using commercial diet for 1 week. After no abnormal symptom was observed, the grass carp was subjected to further study. Then the GCRV challenge experiments and samples collection were conducted as described before [19]. Briefly, viral suspension (4.24×10^6^ TCID_50_/ml) was mixed an equal amount of commercial feed. The resulting feed mixture was used as the source virus. These fish were divided into two groups: treated group and negative control group (n=100 for each group). The fish in the treated group was fed with the feed mixture on the first day, then with commercial feed on the other days. Fish in the negative control group were fed with commercial feed that contained no viral suspension. At 1, 2, 3, 4, 5, and 6 days post infection, five grass carp were collected from each group, respectively. Gill, intestine, liver, spleen, middle kidney and brain samples were isolated from these fish. RNA from these tissues was prepared to analyze the response of *CiBid* to GCRV infection.

Rare minnows were raised and maintained under standard laboratory conditions at China Zebrafish Resource Center (CZRC, http://en.zfish.cn/). Embryos of rare minnow were cultured in 10 cm petri dishes at 28 °C. Adult rare minnows were maintained in the standard tank of an automatic fish housing system. Healthy 4-5 months old adult female and male fish were maintained in separated tanks and mated once a week to obtain progeny. Five mature rare minnows were obtained and RNA from gill, intestine, liver, spleen, middle kidney, head kidney, muscle, heart, and brain were prepared to amplification the cDNA sequence of *GrBid*.

### Detection of apoptosis after GCRV infection

Kidney and spleen tissues were collected from uninfected grass carp (0 days, control) or GCRV infected grass carp collected at different days post infection (1, 3, 5, 7, and 9 days). DNA was extracted from the spleen tissues by DNA Ladder Extraction Kit (Beyotime, China) and subjected to electrophoresis in 1% agarose gel to detect the DNA ladders that induced by GCRV infection. Moreover, TUNEL assay was performed for the kidney that collected above using the In Situ Cell Death Detection Kit (Roche, Switzerland) according to the manufacturer's instructions. Images were observed under a fluoroscope (Olympus MVX10, Japan), and captured using a digital camera (Nikon, MS-SMC, Japan) controlled by ACT-2U software (Nikon, Japan) after diaminobenzidine re-dying.

### Cloning and sequence analysis of grass carp *Bid* (*CiBid*) and rare minnow *Bid* (*GrBid*)

Total RNA was extracted from the tissues of healthy grass carp and rare minnow using Trizol reagent (Invitrogen, USA). The first-strand cDNA synthesis was carried out using DNase I (Promega, USA)-treated total RNA as a template and oligo (dT)-adaptor primer as the control for the reverse transcriptase (TOYOBO, Japan). Specific primers (Table 1) for amplification of *CiBid* and *GrBid* were designed based on the sequences obtained by BLAST analysis sequences of zebrafish *Bid* with the draft genome of grass carp and rare minnow [2, rare minnow genome is unpublished data]. The 5’ and 3’ ends of the *CiBid* and *GrBid* were obtained using the SMARTer^TM^ RACE cDNA Amplification Kit (Invitrogen, USA).

**Table 1:** Primer sequences used in the study

Primers	Sequences (5’ to 3’)	Usage
**CiBid-F**	CACTTCCCTGCTGCTCCTTTCC	CiBid cDNA cloning
**CiBid-R**	AGTTCCTTCTTAAACTCTTTGGCTCCTG	
**GrBid-F**	TCAGTGTTCCCTGCTTCTCC	GrBid cDNA cloning
**CrBid-R**	CCACAGTGCCATAAAGTTTGA	
**CiBidrF**	GGAAGAGGCTCAGGCAGCAAGG	CiBid 3’ RACE
**CiBidrR**	CCCTTGCTGCCTGAGCCTCTTC	CiBid 5’ RACE
**GrBidrF**	CGGTTGGTGAGGAAGAGGC	GrBid 3’ RACE
**GrBidrR**	CCGTCGGTTTGTAATTCTTCG	GrBid 5’ RACE
**CiBid-RTF**	TCCCTGCTGCTCCTTTCC	qRT-PCR for CiBid
**CiBid-RTR**	GTCGGTTTGTAATTCTTCGTCAA	
**gRNA-F**	TGTAATACGACTCACTATAGGAGAAGCAGGG AACACTGGTTTTAGAGCTAGAAATAGC	gRNA amplification
**gRNA-R**	AAAAAAAGCACCGACTCGGTGCCAC	
**TBid-F**	CACCTGTATGAGGCGTTGT	Target site detection
**TBid-R**	ACTATGTCCATCGGTTTGC	
**S6-F**	AGCGCAGCAGGCAATTACTATCT	qRT-PCR for GCRV segment S6
**S6-R**	ATCTGCTGGTAATGCGGAACG	
**GrCaspase-9F**	CGTCCGTCTGGTCATCTATCC	qRT-PCR for GrCaspase-9
**GrCaspase-9R**	GAACTGAGGCAAACCACAATC	
**GrCaspase-3F**	TCGTAATGGGACAGACAGGG	qRT-PCR for GrCaspase-3
**GrCaspase-3R**	GCCATCGGTGCCATAAATC	
**β-RTF**	AGCCATCCTTCTTGGGTATG	qRT-PCR for grass carp β-actin
**β-RTR**	GGTGGGGCGATGATCTTGAT	
**CiBid-RTF**	ACAGAAACGTCAACGTTCCTCA	qRT-PCR for GrBid
**CiBid-RTR**	CTACCTGAGCCTCTACAGCATTGA	
**Grβ-RTF**	TGTAGCCACGCTCGGTCAG	qRT-PCR for rare minnow β-actin
**Grβ-RTR**	GGTATCGTGATGGACTCTGGTG	

The amino acid sequences of CiBid and GrBid were predicted using ORF Finder (http://www.ncbi.nlm.nih.gov/projects/gorf/). The nucleotides and deduced amino acid sequences of *CiBid* and *GrBid* were analyzed using the sequence manipulation suite (http://www.bio-soft.net/sms/index.html). The homologous sequences of CiBid and GrBid from other species were obtained using the BLAST program (http://blast.ncbi.nlm.nih.gov/Blast.cgi). The phylogenetic tree was constructed based on the full-length amino acid sequences of Bid proteins by utilizing MrBayes software (http://mrbayes.sourceforge.net/), and bid amino acid sequences from *Canis lupus*, *Homo sapiens*, and *Mus musculus* were introduced as outgroups.

### Tissue distribution analysis of *CiBid*

Total RNA was extracted from nine tissues (gill, intestine, liver, spleen, middle kidney, head kidney, muscle, heart, and brain) of five healthy grass carp and then reverse-transcribed to obtain cDNA. cDNA from the same tissues were mixed and served as the template for RT-qPCR. Relative expression level of *CiBid* mRNA in different tissues was examined using RT-qPCR in CFX96 real-time PCR detection system (Bio-Rad, USA). The housekeeping gene *β-actin* was used as a reference gene. The specific RT-qPCR primers for *β-actin* and *CiBid* are listed in Table 1. The cycling program for RT-qPCR was as follows: 1 cycle of 95 °C for 2 min, 40 cycles of 95 °C for 15s, 58 °C for 15s, and 72 °C for 30s, followed by dissociation curve analysis to verify the amplification of a single product. All data were depicted as mean ± standard deviation of three replicates. The expression level of *CiBid* was calculated using the 2^-ΔΔCT^ method [41].

### The response of *CiBid* and apoptosis-related genes after GCRV stimulation

Total RNA was extracted from six tissues (gill, intestine, liver, spleen, middle kidney and brain) of five uninfected grass carp or five infected grass carp collected at 1~6 days post infection, and then reverse transcribed to obtain cDNA. cDNA from the same tissues were mixed and served as the template for RT-qPCR. As a mitochondrial pathway initiator, Caspase-9 could activate downstream effectors Caspase-3 and-7 following the stimulation with apoptosis signal [35, 36]. To investigate the impact of GCRV infection on apoptosis *in vivo*, we examined the expression pattern of apoptosis-related genes, including *CiCaspase-9* and *CiCaspase-3*. Primers used in this section were listed in Table 1. The program and reaction mixture for RT-qPCR were the same as above. Data are also shown as mean ± standard deviation of three replicates.

### Cas9 target site design and sgRNA synthesis

We use the CRISPR/Cas9 system to generate *Bid*-deficient (*Bid*^-/-^) rare minnow. The Cas9 target sites of *Bid* were designed with an online tool, ZIFIT Targeter (http://zifit.partners.org/zifit/Introduction.aspx)[42]. pMD19T-gRNA vector, harboring a partial guide RNA sequence, was used in the study [43]. Transcriptional template used for specific sgRNA synthesis was PCR amplified using primers listed in Table 1. sgRNA was transcribed and purified using T7 RNA polymerae (NEB, USA) and Trizol Reagent (Sigma, USA), respectively.

### Cas9 mRNA synthesis

The Cas9 nuclease expression vector pXT7-hCas9 was used for *in vitro* transcription of Cas9 mRNA [43]. First, the vector was linearized by Xbal (NEB, USA). Then, capped Cas9 mRNA was synthesized using mMESSAGE mMACHINE mRNA transcription synthesis kit (Ambion, USA). The Cas9 mRNA was purified using RNeasy Mini Kit (QIAGEN, Germany).

### Microinjection and mutations identification

Cas9 mRNA and sgRNA were co-injected into one-cell stage rare minnow embryos. Approximately 2 nl of solution containing 400ng/μl of Cas9 mRNA and 60ng/μl of sgRNA was injected into each rare minnow embryos. Genomic DNA was isolated from normal developing embryos at 40h after injection for mutations detection. The specific target site was amplified by PCR (primers are listed in Table 1 ). The resulting PCR products were cloned into pMD-18T vector (Takara, Japan), and transformed into competent *Escherichia coli* DH5α cells. Each positive clone was sequenced by a commercial company (TsingKe, China).

### Production of *Bid*-deficient rare minnow and GCRV exposure

After obtaining sexually mature F_0_ rare minnow, we outcross F_0_ fish to a wild-type line. F_1_ embryos were collected and sequenced to confirm those mutations were heritable at 40 hours post-fertilization. Once an ideal lesion was identified, sequenced confirmed F_1_ mutant carriers were incrossed to generate F_2_ stocks, generating 25% homozygous wild-type, 50% heterozygote, and 25% homozygous mutant progeny [44]. The homozygous F_2_ mutant progeny were selected for further study. To confirm the loss of *Bid* expression induced by CRISPR/Cas9 system, seven tissues (gill, spleen, intestine, heart, liver, brain, and muscle) were collected from five *Bid*-deficient rare minnows and wild-type rare minnows. RNA was extracted and then reverse-transcribed to obtain cDNA for RT-qPCR.

For viral challenge experiment, 85 tails of *Bid*-deficient and 81 tails of wild-type rare minnow (2-3g body weight) were firstly soaked in separate tanks containing 6% saline for 2 min. Then, these fish were gathered and immersed in a solution containing GCRV (50ml virus suspension plus 450 ml culture water, titer: 2.97×10^2^ RNA copy/μl) for half an hour. Finally, the fish were transferred to aerated freshwater and cultured at 28 °C.

### Detection of relative copy number of GCRV and apoptosis-related genes

Five individuals were sampled from *Bid*^-/-^ and wild-type groups. Gill, intestine, brain, kidney, and spleen were collected at days before (0 day) and after (1, 2, 3, and 5 days) GCRV exposure. All the samples were used for total RNA isolation and cDNA synthesis. Primers used for detecting copy numbers of GCRV, as well as carrying out the expression level of *GrCaspase-9* and *GrCaspase-3*, were listed in Table 1. *Grβ-actin* was introduced as a reference gene (Table 1). The program and reaction mixture for RT-qPCR were the same as above. Data are also depicted as mean ± standard deviation of three replicates.

### Statistical analysis

The statistical significance between experimental groups and controls was determined by one-way ANOVA and Fisher's least significant difference (LSD) posttest. Differences were considered significant at *p<0.05*. *p*<0.05 was denoted by *.

## SUPPLEMENTARY MATERIALS FIGURES AND TABLES



## Abbreviations

CIK: ctenopharyngodon idellus kidney
cyt c: cytochrome c
CZRC: China Zebrafish Resource Center
dpi: days post infection
dsRNA: double-stranded RNA
GCDC: glychochenodeoxycholate
GCRV: grass carp reovirus
HCV: hepatitis C virus
LPS: lipopolysaccharides
MOMP: mitochondrial outer membrane permeab-ilization
ORF: open reading frame
RACE: rapid-amplification of cDNA ends
RT-qPCR: real-time quantitative PCR
TUNEL: terminal deoxynucleotidyl transferase; dUTP nick end labeling.
