# Supplementary material for: Bid-deficient fish delay grass carp reovirus (GCRV) replication and attenuate GCRV-triggered apoptosis
Source: Oncotarget. 2017 Jul 22;8(44):76408–22. doi: 10.18632/oncotarget.19460 (PMC5652715; doi:10.18632/oncotarget.19460)
Supplement: Supplementary file 1 [file oncotarget-08-76408-s001.pdf]

Bid-deficient fish delay grass carp reovirus (GCRV) replication and attenuate GCRV-triggered apoptosis

SUPPLEMENTARY MATERIALS

```
  1 attgaatgttcattccacagcactttagcgtttgtttgtttgtttgtttttggggataa
 61 tgcttcaccggttttgactgaaagtttaacagcacctttatgaggcattgcatgggtttg
  1     M  D  C  N  R  N  F  N  N  F  Q  H  T  S  L  L  L  L  S
121 tgcaATGGACTGCAATAGAAACTTTAACAATTTTCAGCACACTTCCCTGCTGCTCCTTTC
 20  F  L  E  Q  K  G  C  Q  N  S  E  L  P  E  Q  L  K  L  P  T
181 CTTCCTGGAGCAAAAAGGCTGCCAAAATAGTGAGCTTCCTGAACAGCTAAAGCTGCCTAC
 40  N  H  N  Y  I  E  I  D  E  E  L  Q  T  D  G  H  S  C  S  V
241 CAATCACAATTATATTGAAATTGACGAAGAATTACAAACCGACGGTCACAGTTGCTCAGT
 60  T  Y  R  E  I  L  H  D  L  Q  N  Q  V  Q  P  Q  L  P  V  D
301 CACCTACAGAGAAATTCTGCATGATCTTCAGAATCAAGTTCAGCCTCAACTGCCAGTTGA
 80  E  E  E  A  Q  A  A  R  E  L  A  A  E  L  I  R  I  A  D  L
361 TGAGGAAGAGGCTCAGGCAGCAAGGGAATTGGCTGCAGAATTGATCAGAATTGCAGACCT
100  L  E  Q  R  V  L  F  Q  A  A  E  T  L  T  K  K  L  D  T  C
421 GCTTGAGCAGAGAGTCCTGTTTCAAGCTGCGGAAACTTTGACCAAGAAACTGGACACATG
120  P  T  Q  F  W  A  G  H  L  S  D  G  V  Q  G  L  L  R  Q  V
481 CCCCACACAATTTTGGGCTGGACATCTATCTGATGGAGTGCAGGGCTTGCTTCGCCAGGT
140  A  G  A  K  E  F  K  K  E  L  V  E  M  A  F  T  F  V  L  M
541 GGCAGGAGCCAAAGAGTTTAAGAAGGAACTTGTTGAGATGGCCTTCACTTTTGTGCTCAT
160  K  T  V  C  E  H  V  P  Q  F  L  F  S  L  Y  G  S  V  V  Q
601 GAAGACTGTGTGTGAGCATGTGCCCCAGTTTCTGTTCAGTCTTTATGGCTCAGTGGTCCA
180  Y  F  G  S  R  *
661 GTATTTTGGATCCCGCTGAtccgtgtacaagttatacaattataaggtttagaatgtgaa
721 tgtttgtataaataaaagatatatttgaatgtctttgccttctgtaaaaaaaaaaaaaaa
781 gttgtaaagattgtaaaaaatgaatatccctctcaattgttcatgattattagaacttga
841 agtcatggtcaagactgaaaccctagtgacaccttatgggaatatatattttgttggttt
901 cctttttttaatttttttttcgttactctgttttaatactgtgtgaaacaagcaataaac
961 atgcatttgtgcaaaaaaaaaaaaaa
```

Supplementary Figure 1: Full length cDNA and deduced amino acid sequences of CiBid.

```
  1 tttacgtttgtttttggggagataatgcttcaccggtattttgactgaaagcttacagca
  1                               M  D  C  N  R  N  V  N  V  P
 61 cctgtatgaggcgttgtacaggtttgtgcaATGGACTGCAACAGAAACGTCAACGTTCCT
 11 Q  C  S  L  L  L  L  S  F  F  E  Q  K  G  C  Q  N  S  E  L
121 CAGTGTTCCCTGCTTCTCCTTTCCTTCTTTGAGCAAAAAGGCTGCCAAAATAGTGAGCTT
 31 K  S  E  L  D  T  L  D  E  L  L  K  L  P  T  N  H  T  Y  I
181 AAAAGTGAGCTCGATACTCTTGATGAACTGCTAAAACTGCCTACCAATCACACTTATATT
 51 E  S  D  G  D  L  Q  T  D  G  H  S  F  S  V  T  Y  R  D  L
241 GAGAGTGACGGAGATTTGCAAACCGATGGACATAGTTTCTCTGTCACATACAGAGATCTT
 71 L  H  E  L  Q  N  Q  V  Q  P  Q  L  P  V  N  A  V  E  A  Q
301 TTACATGAACTTCAGAATCAAGTTCAGCCTCAACTGCCAGTCAATGCTGTAGAGGCTCAG
 91 V  A  R  D  M  A  A  E  L  I  R  I  A  D  L  L  E  Q  R  V
361 GTAGCAAGGGATATGGCTGCTGAGTTGATCAGAATTGCAGACCTGCTTGAGCAGAGAGTC
111 L  S  Q  A  A  E  T  L  T  K  K  L  D  T  C  P  T  Q  L  W
421 CTGTCCCAAGCCGCTGAAACTTTGACCAAGAAACTGGACACTTGTCCCACGCAGTTGTGG
131 S  T  H  L  S  E  G  V  Q  G  L  L  H  Q  V  A  G  A  K  E
481 TCCACACATCTATCTGAAGGAGTGCAGGGCTTACTTCACCAGGTGGCCGGAGCCAAAGAA
151 F  K  K  E  L  V  E  M  A  F  T  F  V  L  M  K  T  V  C  E
541 TTTAAGAAGGAACTTGTCGAGATGGCCTTCACTTTTGTGCTCATGAAGACAGTGTGTGAG
171 R  M  P  G  F  L  F  K  L  Y  G  T  V  V  Q  Y  F  G  S  R
601 CGTATGCCCGGGTTTCTGTTCAAACTTTATGGCACTGTGGTGCAGTATTTCGGGTCCCGC
191 *
661 TGAtcccggatgagatcagtgtagaatgtgaatgtttgtataaatatataataatttgaa
721 agtttttgtcttctgtaaaaaaacaaacaaagataaatcctttgtagaagattattcgat
781 cttcatgtcatggtcaagactgaaatccatgtaaccttatgggaattttttttttttttt
841 tttactttttttaatgctttaatactatgtgaaacataataaacattgctttttatgtta
901 aactaaaaaaaaaaaaaaaaaaa
```

Supplementary Figure 2: Full length cDNA and deduced amino acid sequences of GrBid.
